# Paraneoplastic pemphigus associated with chronic lymphocytic leukemia: A case report

**DOI:** 10.1097/MD.0000000000006184

**Published:** 2017-02-24

**Authors:** Qu Jiang, Bin Hong Zhang

**Affiliations:** Department of Hematology, The First Affiliated Hospital of Chongqing University of Medical Sciences, Chongqing, China.

**Keywords:** chronic lymphocytic leukemia, immunosuppressive, infection, paraneoplastic pemphigus

## Abstract

**Rationale:**

Paraneoplastic pemphigus (PNP) is an autoimmune syndrome associated with neoplasms. The treatment approach principally includes suppressing the immunity, but its therapeutic effect is not satisfying.

**Patient concerns:**

We report a case of paraneoplastic pemphigus linked to chronic lymphocytic leukemia in a 63-year-old man.

**Diagnoses:**

At first, the patient was diagnosed with pityriasis rose caused by a viral infection. Biopsies for histology and immunofluorescence showed PNP, was treated with immunosuppressive and antiinfective therapy.

**Interventions:**

Immunosuppressive and antiinfective therapy were performed.

**Outcomes:**

The skin lesions of PNP were alleviated. However, the infections were aggravated and the disease progressed. The patient died of respiratory failure.

**Lessons:**

Treatment for PNP should be adapted to disease severity as early as possible. Antiinfection treatment should be timely and effective because infections are the most common complication that can lead to death.

## 1 Introduction

Paraneoplastic pemphigus (PNP), a rare type of pemphigus associated with malignant diseases, is an autoimmune syndrome that is common in hematological malignancies, such as non-Hodgkin lymphoma, chronic lymphocytic leukemia (CLL), multiple myeloma, and Castleman disease. In addition, it has been reported to occur in association with sarcoma, lung cancer, thymoma, and others.

The etiology may be linked to a humoral immune response and its clinical manifestations involve multiple organs. Common symptoms include refractory stomatitis, oral mucosa erosions, ulcers, and bleeding. Patients can experience severe mucosal damage. Another prominent manifestation is painful and erosive conjunctivitis. Cutaneous lesions are widespread and various, and often show fused erythema, accompanying blisters, erosion, and skin sloughing. The main therapy for PNP is immunosuppression.

## 2 Case report

A man who was born in 1953 was diagnosed with CLL in 2009. He received 6 cycles of chemotherapy with the R-FC regimen (rituximab + fludarabine + cyclophosphamide). Myelograms every 6 months indicated on-going complete remission. In the beginning of 2013, he was diagnosed with sicca syndrome. In March 2015, he was diagnosed with chronic obstructive pulmonary disease based on his history and examinations in our hospital. He had been diagnosed with type II diabetes 5 years prior. A relapse of CLL occurred in March 2015, cytofluorimetric analysis showed that 7% of cells were abnormal monoclonal small B-cell with expressions of CD19, CD20, CD79a, CD22, and sIgMdim. And he got the treatment with one more course of R-FC.

**Figure 1:**
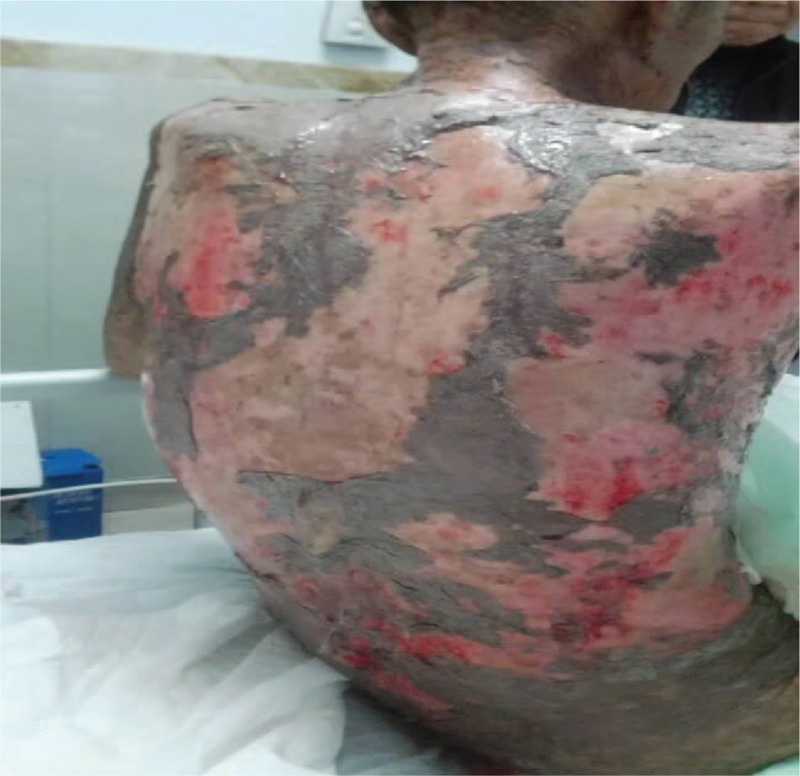
Skin papules and blisters with superficial erosion, accompanied by infections.

**Figure 2:**
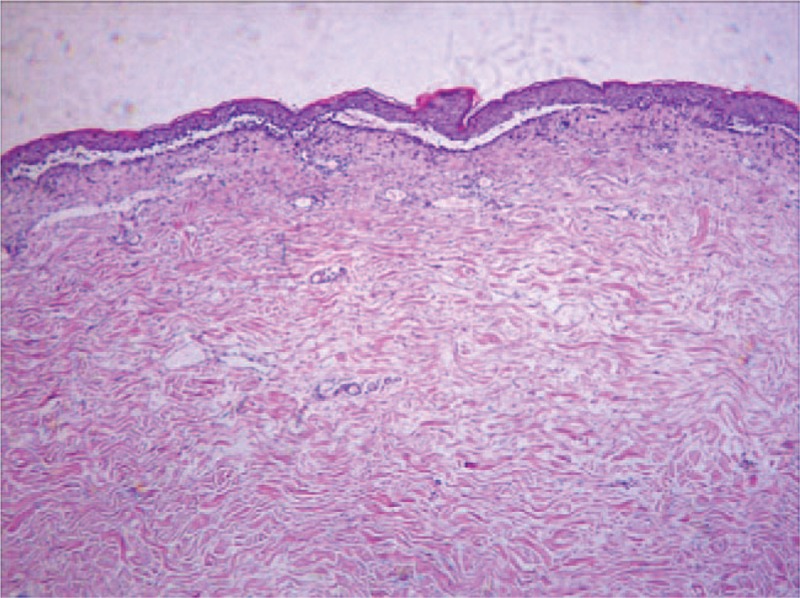
A biopsy specimen from an erythematous lesion.

In February 2016, oral herpes and skin papules and blisters with superficial erosion appeared, accompanied by infections (Fig. 1). A biopsy revealed cleavage within the dermis, epidermal acantholysis, dyskeratotic keratinocytes, vacuolar changes in the layers of the skin, interfacial dermatitis, and epidermal exocytosis (Fig. 2). Immunofluorescence identified intercellular IgG deposits. PNP was considered as the cause.

Panipenem, betamipron, vancomycin hydrochloride, and posaconazole were used to control the infections. Methylprednisolone 40 mg daily, tacrolimus 0.5 mg twice a day, and cyclophosphamide 1000 mg once a day for 2 days were also administered. His condition did not improve and the skin over his entire body was eroded extensively. Treatment was changed to methylprednisolone 40 mg twice a day, cyclosporine 150 mg intravenous continuing for a full 24 hours, and intravenous immunoglobulin 0.4 g/(kg·d) for 3 days. The skin lesions were alleviated by this regimen. However, the infections were aggravated and the disease progressed. The patient died of respiratory failure on April 1, 2016.

## 3 Discussion

PNP is an autoimmune disease that occurs with an underlying lymphoproliferative disorder. The prognosis is poor.^[1]^ Death can occur from a variety of factors, including sepsis, gastrointestinal bleeding, multiorgan failure, and respiratory failure.^[2]^

In this case, the onset of PNP began with erythema, while, in most cases, stomatitis is the earliest symptom. However, at 1st, our patient was diagnosed with pityriasis rosea, which may have been caused by a viral infection. Diagnostic methods included biopsies for histology and immunofluorescence, blood culture and PCR for infectious agents, and serological testing for autoimmunity. Immunoprecipitation is most sensitive method for detecting PNP-specific autoantibodies, and the combined presence of antienvoplakin and antiperiplakin antibodies is most specific and sensitive for PNP detection.^[3]^ In severe or progressing cases, PNP should be considered. It might be difficult to clarify a diagnosis, since clinical findings and histology are not specific. Immunofluorescence micrographs showing presence of intercellular IgG and/or C3 are required for diagnosis, and serological detection of antibodies directed against desmoglein 3 is also necessary.

There is no consensus on the treatment of concurrent PNP and malignant neoplasms.^[2]^ The therapy should aim at controlling the autoimmune process and the underlying malignant disease. There are many case reports on immunosuppressive treatment of PNP in CLL and other lymphomas, using agents such as corticosteroids, cyclosporine, cyclophosphamide, rituximab, alemtuzumab, mycophenolate mofetil, and tacrolimus.^[4–6]^

In this case, methylprednisolone, cyclosporine, tacrolimus, and cyclophosphamide were used to suppress the immune system, and intravenous immunoglobulin was administered to regulate immunity. The CLL was in complete remission since this patient had received FC a year before the onset of PNP, and a relationship could only be conjectured. The treatment with immunosuppressors was slightly effective but was accompanied by more severe infections that were ultimately fatal. In addition, the patient had diabetes and chronic obstructive pulmonary disease, which was in acute exacerbation. He had hypoimmunity and the condition of the diseases worsened rapidly.

Patients with PNP with extensive erythema multiforme-like skin lesions at presentation and histologic keratinocyte necrosis are probably going to have a more severe and rapid fatal outcome because the risk of severe infections is very high.^[7]^ In consideration of a patient's very severe symptoms and the poor prognosis of PNP, we should consider treatment with a severely immunosuppressive agent, like alemtuzumab.^[8]^

In conclusion, when patients with CLL or other neoplastic diseases present with erythema skin lesions or/and herpes labialis the possibility of PNP should be considered and auxiliary examinations should be implemented. Immunosuppressive treatment, which should be adapted to disease severity, should be initiated as early as possible. However, because infections are a common complication that can lead to death, antiinfection treatments should be timely and effective.
